# Field-Dependent Heat Dissipation of Carbon Nanotube Electric Currents

**DOI:** 10.1038/s41598-019-46944-9

**Published:** 2019-07-25

**Authors:** Norvik Voskanian, Eva Olsson, John Cumings

**Affiliations:** 1 Department of Physics, Chalmers University of Technology, 412 96 Gothenburg, Sweden; 2 Department of Materials Science and Engineering, University of Maryland, College Park, Maryland 20740 USA

**Keywords:** Electronic devices, Carbon nanotubes and fullerenes

## Abstract

We study heat dissipation of a multi-wall carbon nanotube (MWCNT) device fabricated from two crossed nanotubes on a SiN_x_ substrate under the influence of a constant (DC) electric bias. By monitoring the temperature of the substrate, we observe negligible Joule heating within the nanotube lattice itself and instead heating occurs in the insulating substrate directly via a remote-scattering heating effect. Using finite element analysis, we estimate a remote heating parameter, *β*, as the ratio of the power dissipated directly in the substrate to the total power applied. The extracted parameters show two distinct bias ranges; a low bias regime where about 85% of the power is dissipated directly into the substrate and a high bias regime where *β* decreases, indicating the onset of traditional Joule heating within the nanotube. Analysis shows that this reduction is consistent with enhanced scattering of charge carriers by optical phonons within the nanotube. The results provide insights into heat dissipation mechanisms of Joule heated nanotube devices that are more complex than a simple heat dissipation mechanism dominated by acoustic phonons, which opens new possibilities for engineering nanoelectronics with improved thermal management.

## Introduction

With the ongoing miniaturization of electronics, operating temperature has become a dominant parameter dictating the efficiency and performance of useful devices^1–3^. There has been significant interest in developing carbon nanotube (CNT) based electronics^4–8^ due to their small size and unique electrical and thermal properties^9,10^. The high crystalline ordering and one-dimensional nature of these materials give rise to many of their features^11^ such as an ability to carry high current densities^3,5,12,13^ and a high thermal conductivity^11^. The practical integration of CNTs in devices requires an in-depth understanding of the influence of all constituent materials on one another, at the nm-scale. The electronic transport of a CNT device will be influenced by its contact resistance^14^ as well as its interaction with its supporting substrate^15,16^. Similarly, the operating temperature of the device will depend on the heat dissipation mechanism and efficiency of all the components to carry the heat away.

The Kapitza resistance observed in nanotubes^17,18^ can reduce the effective thermal conductivity of overall devices and, together with a high current density, can result in overheating^19,20^, posing a major thermal management challenge. Ideally, the nanotube should have a highly efficient heat dissipation channel in order to operate as an effective current carrier. Both experimental^21^ and theoretical^22^ work have indicated the presence of a highly efficient heat transport mechanism in current-carrying CNT devices. This process provides an energy dissipation channel for the hot electrons into the supporting substrate and diminishes the excitation of the CNT optical phonons^22^. As a result, energetic electrons heat up the supporting substrate and avoid the direct heating of the current-carrying nanotube. In this letter, we demonstrate further evidence for the presence of this dominant heat dissipation mechanism for a nanotube device fabricated on a SiN_x_ substrate. Our observations additionally demonstrate a clear field-dependence of this dissipation mechanism. We have quantified the strength of the process and have evaluated its behavior as a function of applied bias. The results indicate a highly efficient dissipation at low biases where more than 80% of the power is directly dissipated into the substrate. At higher biases the dominance of this transport channel is reduced as electronic heating is increased, leading to gradual excitation of the optical phonon modes of the CNT.

In short channels, the unique 1D nature and band structure of nanotubes can lead to a ballistic electron transport mechanism. For nanotubes longer than the charge-carrier mean free path, the mechanism becomes diffusive resulting eventually in increased inelastic scattering processes, where optical phonon energies become accessible^23,24^. This diffusive transport is believed to result in substantial heating of the CNT, due to the excitation of on-tube optical phonons^25^ and has been shown by a variety of thermal measurement techniques^26,27^. A Joule-heated nanotube quickly reaches a thermal equilibrium state along its length due to its high thermal conductivity, associated with a high degree of lattice structural ordering and strong sp^2^ bonding^28^. Both theoretical and experimental work have shown the nanotube thermal conductivity^29^, *k*_*CNT*_, to be on the order of magnitude of 1000 W.m^−1^.K^−1 ^^30^, even larger than traditional high *k* metals.

The temperature distribution of the CNT, after reaching a thermal equilibrium, is governed by heat dissipation to the environment. This is typically dominated by phonon conduction, and these phonon-phonon interactions at the interface can dictate the operational device temperature, quantified by the interfacial thermal boundary resistance (TBR)^31,32^. In a low pressure environment there is no convective transport and the thermal exchange via far-field black body radiation (Stefan-Boltzmann law) is negligible, producing less than 1 K deviation even when included in the modeling results presented below. Therefore, tuning the TBR or introducing other mechanisms of heat dissipation can significantly affect the nanotube temperature and influence device performance. However, in the case of CNTs biased on certain substrates, the interaction of the hot charge carriers with the substrate’s optical phonons or phonon-polaritons has been shown to provide a dominant means of heat dissipation and diminishing Joule heating within the CNT lattice^21,22^. Complete understanding of these highly efficient modes of heat dissipation can uncover new techniques in designing cool electronics and provide solutions to thermal management issues.

## Results and Discussions

In this study, the heat dissipation mechanism of current carrying MWCNTs on supported SiN_x_ membranes is investigated using Electron Thermal Microscopy (EThM)^33^, a technique based on observing the solid to liquid phase transition of Indium (In) islands. Usually, the TBR between the nanotube and the substrate dictates the amount of thermal energy transferred between the materials. However, we observe direct evidence of a dominant heat dissipation mechanism which heats the substrate above 429 K, the melting point of In. In addition, the experimental setup facilitates exploring field dependent thermal transport in a crossed MWCNTs structure by allowing to selectively bias the device. By monitoring the temperature of the substrate we observe heating concentrated only under the current carrying segments of the MWCNTs with no evidence of heating below the non-biased region of the crossed nanotubes. We quantify the extent of this remote substrate heating and demonstrate its decreasing nature in response to increased applied voltage.

**Figure 1:**
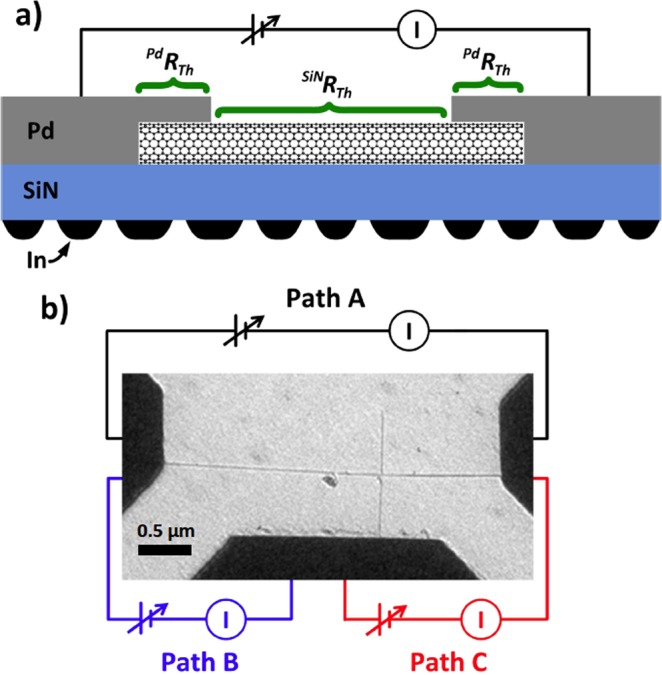
(**a**) Side view schematic of a nanotube device. (**b**) Bright field TEM image of the crossed MWCNT device with the color coded circuit indicating the 3 different measurement conditions.

Figure 1(a) shows a profile schematic of a typical device with the MWCNTs on the supporting SiN_x_ membrane. The samples are fabricated on commercially available 50 nm thick SiN_x_ membranes supported on 200 nm thick silicon substrate with a 250 μm × 250 μm etched region. The electron transparent SiN_x_ is suspended in this region allowing for TEM experiments to be carried out. First, using e-beam lithography and metal deposition, a series of alignment markers are patterned on the membrane. Next, the arc-discharge-synthesized MWCNTs, from Sigma Aldrich, are placed in an IPA solution and spun cast at 4000 rpm, and are in turn characterized by a TEM with 100 kV acceleration voltage. By observing the relative location of the desired nanotube to the alignment markers another lithography step is carried out to pattern the Pd electrodes, allowing to carry out Joule heating experiments. As a final step, 20 nm of In metal is thermally evaporated on the backside of the sample, producing islands that act as binary thermometers. More detailed description of the fabrication process and the tools used can be found in prior reports^21,33,34^.

Figure 1(b) shows a bright field TEM image of a crossed MWCNT device, before the In deposition, with the three current paths. The crossed MWCNTs device allows to either bias the horizontal nanotube, 31 nm in diameter, using the side electrodes (black, Path A) or to run the current from horizontal nanotube into the vertical one, 24 nm in diameter, in either the long cross (blue, Path B) or short cross (red, Path C) configuration. An important advantage of the designed geometry is that it allows to monitor heat dissipation of a single MWCNT where only a specific section is placed under an electric field. Using a biasing TEM holder the contact pads are connected to an external DC power supply (Keithley 236) allowing for *in situ* TEM experiments, with the third contact left electrically floating. By operating the TEM in the appropriate dark field condition it is possible to observe a contrast difference between the solid and molten In islands due to Bragg diffraction, explained in more detail previously^33^. The melting point of In, at 429 K is independent of size for islands above 50 nm and thus makes it possible to use this technique to visualize the temperature distribution across the SiN_x_. The MWCNT heat dissipation via conduction to the substrate strongly depends on the interfacial TBR, *R*_*c*_, and the thermal conductivity, *k*, of the incorporated material. This dependence can be seen in terms of the heat diffusion equation,

1 $$\nabla \cdot ({\rm{k}}\,\nabla T)+Q-\frac{{\rm{\Delta }}T}{{R}_{c}}=0,$$

where *k* and Δ*T* are the lattice thermal conductivity and temperature gradient across the interface, *Q* is the heat source and *R*_*c*_ is the *TBR* at the interface.

It has been shown^29,34–36^ that there can be a large TBR between a MWCNT and its supported substrate ^*SiN*^*R*_*c*_, on order of 250 K.m W^−1^ [((kelvin)(meter))/(watts)], due to the substrate surface roughness and resulting small contact area. Thermal boundary conduction requires intimate atomic contact and even small amounts of roughness can greatly increase *R*_*c*._ However, coating the nanotube with metal can drastically increase the contact area to reduce the TBR, ^*Pd*^*R*_*c*_, to 4.2 K.m W^−1^. Therefore, phonon mediated heat conduction from the nanotube to the substrate is highest in the metal covered regions.

**Figure 2:**
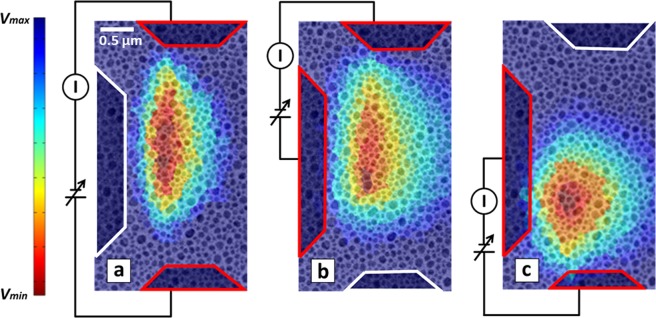
Experimental color maps overlaid on TEM images demonstrating the temperature gradient across the SiN_x_ membrane for the 3 current paths. Images depict the same region as Fig. 1b.

In the EThM measurement technique, the melting of the In islands provides a real-time thermal map of the entire device with nanoscale resolution. In particular, the location of the earliest-melting islands reflects the location of optimal thermal coupling between the nanotube and the membrane. For each image, the voltage and current are stabilized quickly, within one second, and then images are recorded with 10 second exposure, where the current fluctuations are less than 1%. To best visualize the thermal map over the entire voltage range, a unique color is assigned to each molten island based on its melting voltage and an experimental color map is generated, as seen in Fig. 2, with red assigned to the initial molten islands. From Fig. 2a, it can be seen that the initial melting occurs in the middle of the device, at the midpoint of the biased MWCNT furthest from the contacts. The position of the hotspot contradicts previous observation of poor thermal coupling between the nanotube and the SiN_x_, based on the high ^*SiN*^*R*_*c*_ values. These results indicate the presence of a much more efficient mode of heat dissipation for the case of biased MWCNTs, which has been observed in other devices^21,37^, referred to as *remote Joule heating*.

Furthermore, Fig. 2b,c clearly show that the SiN_x_ heats up only under the current carrying region of the MWCNTs. This is of particular interest, especially when considering the high thermal conductivity of CNTs (*k*_*CNT*_) in the range of 1000–3000 Wm^−1^K^−1 ^^29^ In a Joule heated CNT device, the high *k*_*CNT*_, will result in efficient heat propagation within the nanotube lattice achieving quick steady-state thermal equilibrium. Therefore, the lack of substrate heating in the unbiased region of the device suggests that the nanotube is much colder than the 429 K required to melt the In. Put simply, the high thermal conductivity of the CNT and low thermal conductance of the interfaces would predict that all three current paths should give the same heating profile for simple phonon-mediated conduction. The confinement of the molten islands to the current carrying region indicates that the hot charge carriers instead dissipate energy directly to the substrate. The underlying cause of this phenomenon may be due to the excitation of polarizable modes within the SiN_x_ membrane, such as optical phonons or surface phonon polaritons^22^, which in turn decay into the traditional acoustic phonon modes in the SiN_x_, heating the substrate. Moreover, the white border in Fig. 2 indicates a floating electrode and demonstrates the current-induced nature of this remote heating mechanism which is completely suppressed at zero current.

To quantify the magnitude of the power dissipation into the substrate the devices are simulated using finite element analysis, COMSOL software package, based on a modified heat conduction equation where the nanotube system is treated by

2 $$\nabla \cdot ({\rm{k}}\,\nabla T)+(1-\beta )Q-\frac{({T}_{CNT}-{T}_{SiN})}{{R}_{c}}=0$$

and the SiN substrate by

3 $$\nabla \cdot ({\rm{k}}\,\nabla T)+\beta Q-\frac{({T}_{SiN}-{T}_{CNT})}{{R}_{c}}=0$$

where *β*, the remote heating parameter, is the ratio of the power dissipated into the substrate over the total power applied. The thermal conductivity of the substrate *k*_*SiN*_ (3.5 Wm^−1^K^−1^)^33^ and the *R*_*c*_ values^34^ have been measured independently in a series of controlled experiments, described in previous publications and confirmed by numerous similar measurements conducted by us. The electrical properties of the metal have been independently measured using Van der Pauw technique and their thermal conductivities have been calculated based on the Wiedemann-Franz law. A *k*_*CNT*_ value of 1000 Wm^−1^K^−1^ has been used in the simulation based on literature reported values and our own observations. The electrical resistivity of the CNT, *ρ*_*CNT*_, and the *β* value have been extracted from the simulations by an iterative procedure, described in detail in the Supplementary Information. In brief, for a given applied potential, the *ρ*_*CNT*_ value is varied to match the experimental current density calculated based on the IV data from the power supply. Next, the β value is varied to match the experimental melting profile observed for the same applied potential. This process is repeated iteratively until a unique *ρ*_*CNT*_(*V)* and *β*(*V)* is extracted for each voltage. For more details of the modeling and analysis, see the Supporting Information document.

**Figure 3:**
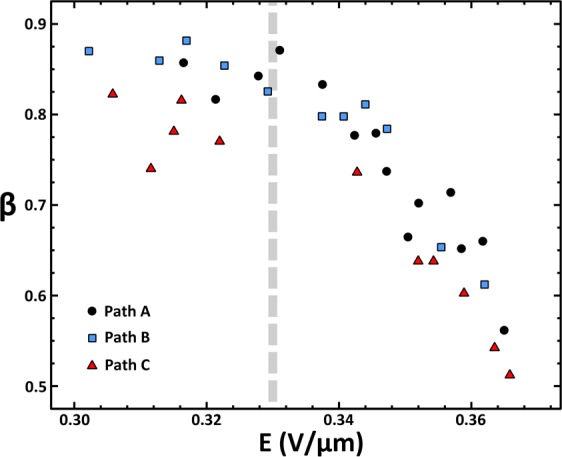
Extracted *β* values as a function of electric field at the center of the nanotube channel. The vertical dashed line indicates the field where charge carriers gain enough energy between scattering events to reach on-CNT optical phonons, reducing the substrate remote heating effect.

Figure 3 shows the results of the simulations for the three configurations. Here, *β* is plotted vs. the electric field in the nanotube channel, which we will come back to below. Figure 3 shows that 80% to 86% of the power is directly dissipated into the substrate for the lowest applied biases, consistent with previous reports^21^. The high value of β indicates the direct generation of substrate excitations by the hot charge carriers as the dominant mechanism of substrate heating, supporting the remote heating model, where only a small fraction of the dissipation finds its way into the nanotube acoustic and optical phonons. This is due in part to the longer range of the remote heating effect^15^, which can span the gap between the CNT and substrate due to the SiN_x_ surface roughness of approximately 0.8 nm RMS. Direct Joule heating of the nanotube itself instead would require the excitation of the high energy CNT optical phonons, at about 161–200 meV^4,13^. However, the availability of lower energy optical phonons supported by the SiN_x_, at about 92–121 meV^38^, provides a competitive pathway for energy dissipation of the hot charge carriers directly into substrate modes. This results in substantial heating of the membrane as the substrate optical phonon modes dissipate into bulk modes, producing a remote heating effect. A similar process has been invoked to explain heat dissipation in CNT devices on polar substrates by excitation of substrate surface phonon polaritons^22^, and it has also been shown to play a direct role in current saturation in graphene devices^15,39,40^. We note a vertical spread of data in Fig. 3, that is indicative of the error in the experimental beta determination resulting from our algorithm. Systematic error in the algorithm, from e.g. current measurements or in the location of the islands produces errors that are small, comparable to the size of the data points. Thus these errors are inherent, of unknown origin. Nevertheless, the trend toward smaller beta at higher electric field emerges as a reproducible and significant experimental result, if it could be connected with a explanatory model, which we now will discuss as follows.

From the data in Fig. 3, it is apparent that the remote heating of the substrate has two distinct trends over the range of applied field. At lower field, there is no observable systematic change in *β*, but as the power is increased, at fields above 0.33 V/μm, the *β* value decreases precipitously. By comparing resistance measurements along the three different current paths with the corresponding path lengths, we estimate the resistance per length ρ of the CNT as 17.7 kΩ/μm, as an upper bound. The total contact resistances can similarly be estimated as 8.8 kΩ or less, which is negligible for these studies. Using Datta’s diffusive *elastic resistor* model^41^, the mean elastic free path can be estimated as

4 $$\lambda =\frac{h}{{q}^{2}}\frac{1}{M\rho },$$

where h is Planck’s constant, q is the charge on an electron, ρ is the resistance per length, and M is the number of quantum modes in the conducting channel. For multiwall carbon nanotubes, the layers may have differing electronic properties and the low inter-tube conductivity can be much lower than the conductivity within individual tubes^42,43^. As described in ref.^43^, the current in an externally contacted nanotube starts in the outermost layer, transitioning to inner layers over a length scale of about ~1 μm per layer, which would give about three layers participating in the current flow for the measurements reported here. Each shell has an orbital degeneracy of two and a spin degeneracy of two, and thus we can estimate M = 3 × 2 × 2 = 12, giving a mean free path of λ = 121 nm. The product of the mean free path and the electric fields probed here give energies in the range of 36–44 meV, indicating that carriers do not gain sufficient energy between successive scattering events for dissipative inelastic scattering to optical phonons in either the SiN substrate or the CNT itself. Thus, the measured nanotube resistance is dominated by elastic scattering^44^, and carriers arrive at successive dissipation events through multiple elastic scattering and a process of diffusion, both consistent with the elastic resistor model. In this process, inelastic scattering from nanotube acoustic phonons is inefficient^13^ and carriers build energy diffusing in the electric field until enough kinetic energy is available for dissipation to the lowest available mode with sufficient coupling, namely substrate optical phonons at 92–121 meV. This drives the remote Joule heating effect at the lower biases in the present study. When individual scattering events provide enough energy also to reach the nanotube optical phonons at higher energy, then such dissipation will increase, resulting in a decrease of the remote heating effect. This will occur at a field which spans the gap from the highest energy Si-N vibration at about 975 cm^−1^ (121 meV) to the lowest CNT optical phonon at 1300 cm^−1^ (161 meV)^13^, with an additional energy per scattering event of ΔE = 40 meV. This predicts a transition field of E_T_ = ΔE/eλ, as indicated by the vertical line in Fig. 3, which agrees with our observed downturn in β.

In summary, by monitoring the temperature gradient across the supporting SiN_x_ membrane we have demonstrated a highly effective substrate heating process. This mechanism is due to the interaction of hot charge carriers within the supported CNT with the optical modes of the SiN_x_. The concentrated heating of the substrate within the current carrying region of the CNT is a direct indication of the relatively low acoustic phonon temperature of the CNT and therefore the absence of a conventional Joule heating process. The simulations have allowed to quantify the amount of the remote heating observed and show its dependence on the electric field. Further development of this heat transport model could be used in designing electronic interconnects with improved thermal management, which could be critical in future device applications.

## Methods

### Device fabrication

Samples were fabricated on free-standing 50 nm SiN membranes in a multi-step process. Initially, using electron beam lithography, metal deposition and liftoff procedure 30 nm of Cr/Au alignment markers and large electrical contact pads are patterned on the substrate. Next, MWCNTs grown by arc-discharge (Sigma Aldrich) are spin casted from an isopropanol suspension and their quality and position, relative to the alignment markers, is evaluated using TEM at 80 kV to avoid beam damage. To complete the circuit the sample is once again patterned using e-beam lithography by depositing Pd to connect the large Cr/Au pads to the MWCNT. As a final step, using thermal evaporation In is deposited on the back side of the substrate.

### Thermometry

Using a biasing holder the thermal measurements were performed in a JEOL JEM-2100 TEM in the dark-field condition to provide contrast difference between solid and molten In islands. Using an external power supply the voltage was increased and a new image of the molten In islands was captured. By assigning a unique color to the islands molten at each given voltage thermal maps were generated indicating the thermal profile of the device.

## Supplementary information

Supporting Information: Field-Dependent Heat Dissipation of Nanotube Electric Currents

## Data Availability

The datasets generated during and/or analysed during the current study are available from the corresponding author on reasonable request.
