# Supplementary material for: Field-Dependent Heat Dissipation of Carbon Nanotube Electric Currents
Source: Sci Rep. 2019 Jul 25;9:10785. doi: 10.1038/s41598-019-46944-9 (PMC6658496; doi:10.1038/s41598-019-46944-9)
Supplement: Supplementary file 1 — Supporting Information: Field-Dependent Heat Dissipation of Nanotube Electric Currents [file 41598_2019_46944_MOESM1_ESM.pdf]

Supporting Information: Field-Dependent Heat Dissipation of Nanotube Electric Currents

Norvik Voskanian, †, ‡ Eva Olsson† and John Cumings,‡*

†Department of Physics, Chalmers University of Technology, Gothenburg, Sweden. ‡Department of Materials Science and Engineering, University of Maryland, College Park, Maryland 20740, USA.
Correspondence and requests for materials should be addressed to J. C. (email: cumings@umd.edu)

Simulation Procedure

We use COMSOL, a finite elemental analysis software, to simulate the experiments and reach a quantitative understanding of the heat dissipation mechanism by solving the modified heat equation (2). The device was characterized with a TEM at low acceleration voltage to obtain accurate dimensions of the nanotubes and the electrodes and their position relative to each other. The model includes the entire 250 μm x 250 μm suspended 50nm thick SiN_x with the edges set at 293 K (T_o). The much larger silicon supported region is excluded to reduce simulation time since there is negligible temperature gradient due to its large thickness and strong thermal coupling to the TEM holder. Figure S1 shows the relative dimensions of the suspended SiN region (the window in the center of the device) compared to the entire device supported on 200 μm thick silicon substrate (outlined by the red dashed line) justifying the omission of the substrate and the TEM holder in the thermal model. In addition, a mesh convergence test was carried out to optimize the meshing parameters for the model.

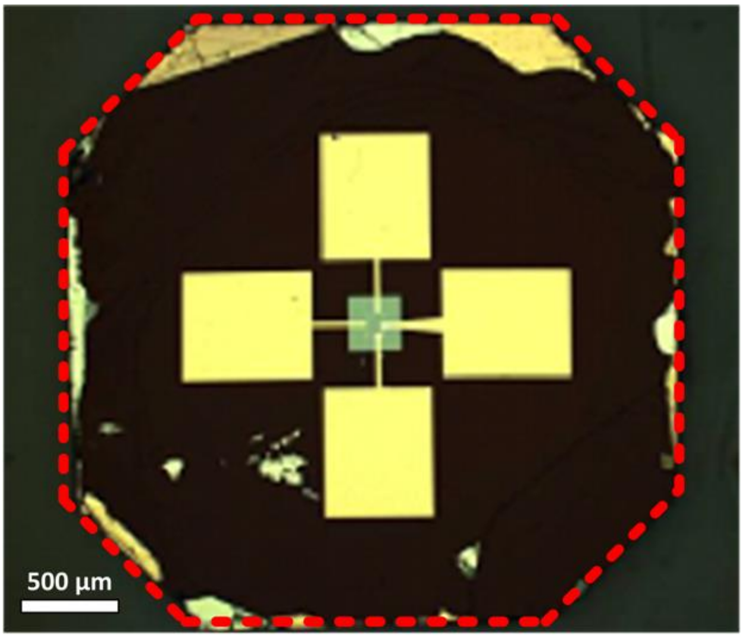

Figure S1. An optical image of a typical device with a 250 µm x 250 µm suspended SiN membrane at the center and the 4 contact electrodes for biasing experiments.

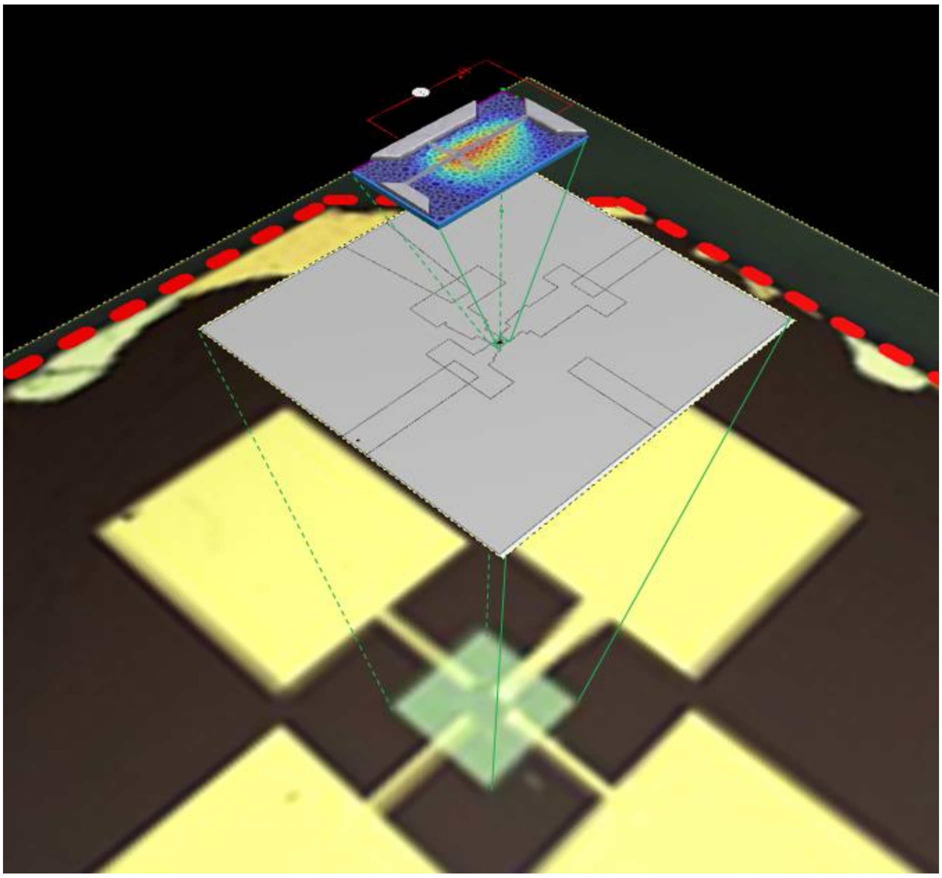

Figure S2. The simulated region indicated by the green lines. Including the nanotube, electrodes and the suspended SiN_x.

Most of the electrical and thermal properties of the material were obtained from previous controlled experiments[1, 2]. However, the remote heating parameter and the electrical resistivity of the nanotube, β and ρ respectively, were set as free parameters to match the experimental observations. For each applied voltage the current density, J_{exp}, was calculated from the IV curve which was checked in the simulation, J_{sim}. By matching the J_{sim} to J_{exp} to within 3% an optimum ρ value was extracted for each bias. Similarly, the β values were extracted by matching the observed melting profile from the dark field images. Based on calibrated experiments using a dedicated heating holder the melting point of the islands is measured to be at 429K which indicates the temperature of the substrate at the edge of the molten region. Consequently, in the simulations β was varied until the substrate temperature corresponded to the observed melting profile for each given voltage, Figure S3. The process of finding the correct β and ρ was performed iteratively, Figure S4, to find a single convergent solution the results of which are shown in Figure 3. The iterations were automated by MATLAB.

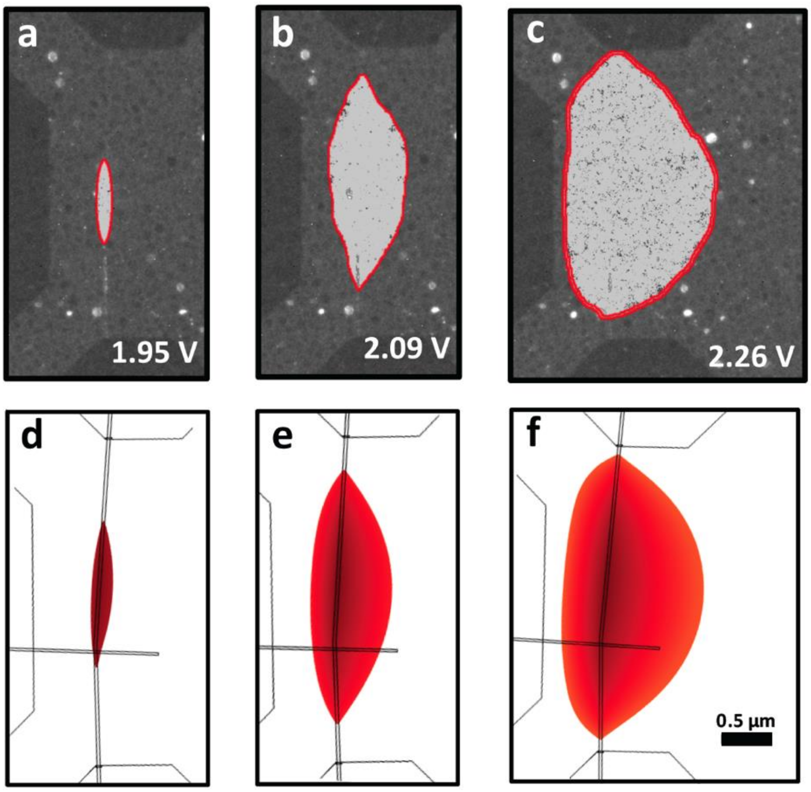

Figure S3. Experimental DF images collected at 3 different voltages demonstrating the area of molten islands and their corresponding simulated results from finite element package.

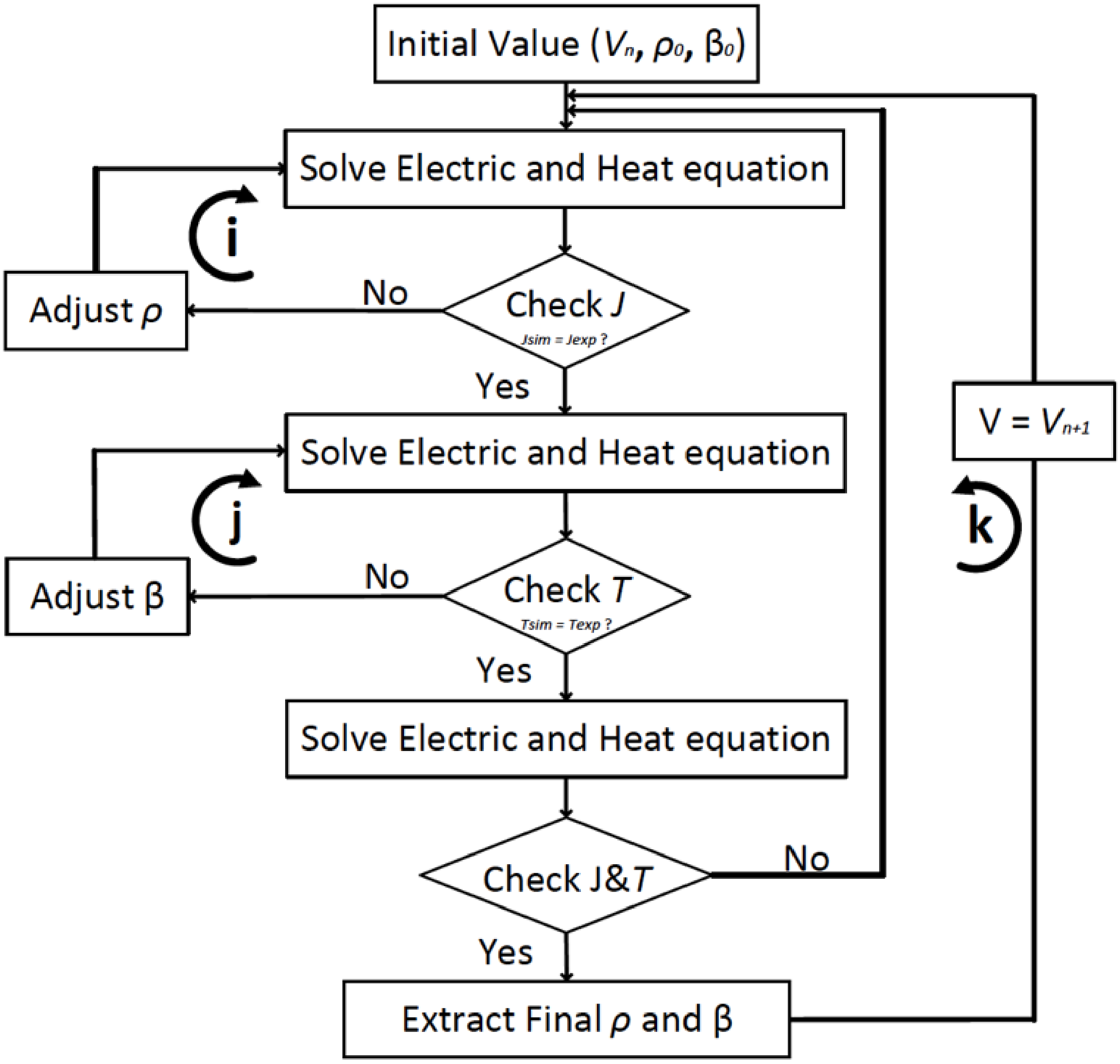

Figure S4. Block diagram of the iterative process used to extract the β and ρ for each voltage.

2D model

The simulations are based on a two-dimensional model of the device due to the large variation in the dimensions of the constituting elements in the model and the time associated with a single convergence process. However, we have previously reported the validity of the 2D vs. the 3D model for a similar device[3] (see supplementary information therein, section 3.4).

The thermal model is based on solving the steady-state heat diffusion equation

$$\nabla \cdot (\mathrm{k}\,\nabla T) + Q = 0$$

Where k is the thermal conductivity of the element and Q is the heat source. In a more detailed overview of the simulation, as seen in Figure S5, the temperature gradient and the heat source for each element have been outlined. The 2D model uses three different interconnected layers which are solved together in each iteration step. The SiN layer, responsible for calculating the temperature of the substrate (T_{SiN}) is assigned to the substrate with the k designated to the SiN thermal conductivity (k_{SiN}). In addition, different TBRs are assigned to the model depending on the particular material as well as its interaction with the other layers, dictating the designated temperature gradient and the boundary conditions. The two other layers are responsible for the temperature of the metal electrodes, T_{metal}, and the temperature of the nanotube, T_{CNT}. As described above, the heat source Q is based on the IV measurements from the power supply. However, to model the remote heating the heat source is incorporated in terms of the β parameter. For the metal layer, the heat source for the electrodes and the nanotube sections covered by the Pd is described by $Q - \frac{(T_{metal} - T_{SiN})}{R_{th}}$ and $Q - \frac{(T_{metal} - T_{CNT})}{R_{cPd}} - \frac{(T_{metal} - T_{SiN})}{R_{th}}$ respectively, where R_{th} is the TBR for the Pd-SiN interface and R_{cPd} for the Pd covered CNT-SiN interface. On the other hand the nanotube region between the electrodes, supported on the membrane, is defined by $(\beta) * Q - \frac{(T_{SiN} - T_{CNT})}{R_{cSiN}}$ in the SiN layer and $(1 - \beta) * Q - \frac{(T_{CNT} - T_{SiN})}{R_{cSiN}}$ in the nanotube layer. As such a single iteration step, as presented in Figure S4, produces a solution to T_{metal}, T_{CNT}, and T_{SiN} for a given set of initial conditions.

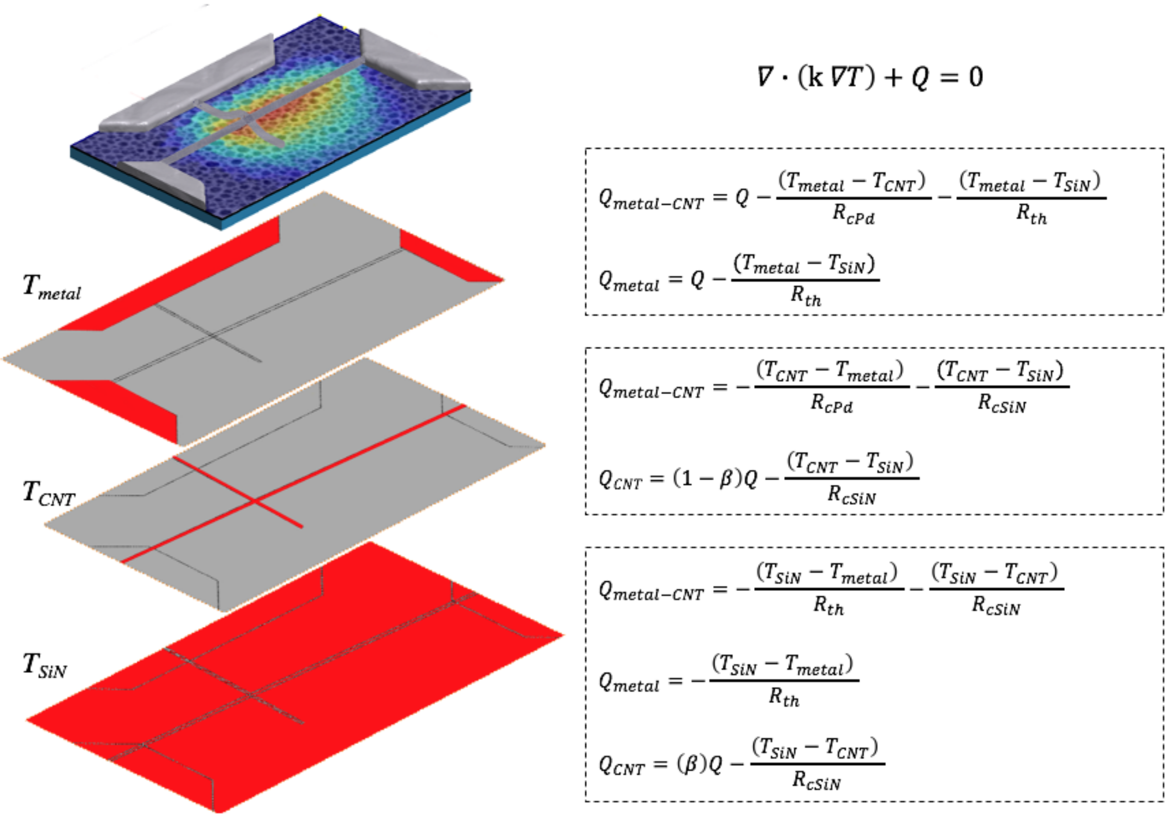

Figure S5. Simulation process with 3 thermal models designated to the substrate temperature (T_{SiN}), CNT temperature (T_{CNT}), and the temperature of the electrodes (T_{metal}). The corresponding heat source, Q, for each layer is defined in the dashed boxes with $Q_{metal\text{-}CNT}$ designated to the region where the CNT is coated with the Pd metal.

The decision to use 1000 $Wm^{-1}K^{-1}$ as the thermal conductivity of the nanotube (k_{CNT}) was based on testing the effects of k_{CNT} on the remote heating parameter (β). It was observed that with k_{CNT} = 3000 $Wm^{-1}K^{-1}$ the beta value changes by less than 0.01 (>2%) at large fields and less than 0.005 at the lower field regime. Therefore, the most conservative and realistic value of 1000 $Wm^{-1}K^{-1}$ was chosen for the analysis to highlight the importance of the remote heating phenomena.

REFERENCES:

1. Baloch, K. H.; Voskanian, N.; Cumings, J., Controlling the thermal contact resistance of a carbon nanotube heat spreader. *Applied Physics Letters* **2010,** *97* (6).

2. Brintlinger, T.; Qi, Y.; Baloch, K. H.; Goldhaber-Gordon, D.; Cumings, J., Electron thermal microscopy. *Nano Letters* **2008,** *8* (2), 582-585.
3. Baloch, K. H.; Voskanian, N.; Bronsgeest, M.; Cumings, J., Remote Joule heating by a carbon nanotube. *Nature Nanotechnology* **2012,** *7* (5), 315-318.
